# Low prevalence of *Plasmodium malariae* and *Plasmodium ovale* mono-infections among children in the Democratic Republic of the Congo: a population-based, cross-sectional study

**DOI:** 10.1186/s12936-016-1409-0

**Published:** 2016-07-08

**Authors:** Stephanie M. Doctor, Yunhao Liu, Olivia G. Anderson, Amy N. Whitesell, Melchior Kashamuka Mwandagalirwa, Jérémie Muwonga, Corinna Keeler, Michael Emch, Joris L. Likwela, Antoinette Tshefu, Steven R. Meshnick

**Affiliations:** Department of Epidemiology, Gillings School of Global Public Health, University of North Carolina, Chapel Hill, NC 27599-7435 USA; Department of Statistics and Operations Research, University of North Carolina, Chapel Hill, NC 27599 USA; Ecole de Santé Publique, Faculté de Medecine, Université de Kinshasa, Kinshasa, Democratic Republic of the Congo; Programme National de Lutte contre le SIDA et les IST, Kinshasa, Democratic Republic of the Congo; Department of Geography, University of North Carolina, Chapel Hill, NC 27599 USA; Programme National de Lutte contre le Paludisme, Kinshasa, Democratic Republic of the Congo

**Keywords:** *Plasmodium malariae*, *Plasmodium ovale*, Democratic Republic of the Congo, Rapid diagnostic test

## Abstract

**Background:**

In an effort to improve surveillance for epidemiological and clinical outcomes, rapid diagnostic tests (RDTs) have become increasingly widespread as cost-effective and field-ready methods of malaria diagnosis. However, there are concerns that using RDTs specific to *Plasmodium falciparum* may lead to missed detection of other malaria species such as *Plasmodium malariae* and *Plasmodium ovale*.

**Methods:**

Four hundred and sixty six samples were selected from children under 5 years old in the Democratic Republic of the Congo (DRC) who took part in a Demographic and Health Survey (DHS) in 2013–14. These samples were first tested for all *Plasmodium* species using an 18S ribosomal RNA-targeted real-time PCR; malaria-positive samples were then tested for *P. falciparum, P. malariae* and *P.ovale* using a highly sensitive nested PCR.

**Results:**

The prevalence of *P. falciparum, P. malariae* and *P. ovale* were 46.6, 12.9 and 8.3 %, respectively. Most *P. malariae* and *P. ovale* infections were co-infected with *P. falciparum*—the prevalence of mono-infections of these species were only 1.0 and 0.6 %, respectively. Six out of these eight mono-infections were negative by RDT. The prevalence of *P. falciparum* by the more sensitive nested PCR was higher than that found previously by real-time PCR.

**Conclusions:**

*Plasmodium malariae* and *P. ovale* remain endemic at a low rate in the DRC, but the risk of missing malarial infections of these species due to falciparum-specific RDT use is low. The observed prevalence of *P. falciparum* is higher with a more sensitive PCR method.

**Electronic supplementary material:**

The online version of this article (doi:10.1186/s12936-016-1409-0) contains supplementary material, which is available to authorized users.

## Background

Malaria remains a severe global burden, causing an estimated 214 million cases and 438,000 deaths in 2015 [1], a toll that is particularly high in sub-Saharan Africa. Efforts to control malaria depend on diagnostic accuracy and availability, and in recent years the demand for rapid diagnostic tests (RDTs) has been increasingly high. RDTs detect malaria antigens in the blood using immunochromatography. They provide an easy-to-use alternative to microscopy, which requires skilled experts to be optimally effective [2, 3], and a cost-effective and field-ready alternative to PCR, which is more sensitive [4] but requires expensive equipment.

Among the most widely used RDTs are those that detect *Plasmodium falciparum* histidine-rich protein 2 (*Pf*HRP2). These RDTs are sensitive and specific for parasitaemias above 200 per µl blood [5]. RDTs that use the *Plasmodium* lactate dehydrogenase (pLDH) antigen are designed to detect all species of malaria, but are less sensitive [6–8].

In sub-Saharan Africa, where the vast majority of malaria infections are due to *P. falciparum* [1], RDTs detecting *Pf*HRP2 are most commonly used. However, mono-infections with non-falciparum malarias (primarily *Plasmodium malariae* and *Plasmodium ovale* in sub-Saharan Africa) may go undetected. In this study, a sub-set of samples from a nationally representative, cross-sectional study of children under age 5 years in the Democratic Republic of the Congo (DRC) were used to determine the prevalence of *P. malariae* and *P. ovale* mixed and mono-infections, and to assess the risk of missed detection due to the use of falciparum-specific RDTs.

## Methods

### Survey methodology and sample collection

The 2013–14 Demographic and Health Survey (DHS) was a cluster-based household survey in the DRC, which took place between November 2013 and February 2014. As part of the survey, blood samples collected from children under 5 years of age were analysed for malaria infection, without speciation, by light microscopy. The samples were also tested with an RDT targeting *Pf*HRP2 (SD Bioline Malaria Ag P.f., Standard Diagnostics, Gyeonggi-do, Republic of Korea) and used to make dried blood spots (DBS). From DBS, DNA was extracted and tested for *P. falciparum* infection using a real-time PCR assay targeting the *P. falciparum* lactate dehydrogenase (*pfldh*) gene as previously described [9, 10]. This research was approved by institutional review boards at the Kinshasa School of Public Health and the University of North Carolina at Chapel Hill. Informed consent was obtained from a parent or responsible adult for all subjects.

Samples for this study were randomly chosen from four strata: (1) microscopy-positive, *pfldh* PCR-negative; (2) microscopy-negative, *pfldh* PCR-negative; (3) microscopy-positive, *pfldh* PCR-positive; and, (4) microscopy-negative, *pfldh* PCR-positive (Table 1). To detect a prevalence of 1 % (0, 2, 95 % confidence interval), the minimum sample size was estimated as 362 [11].

**Table 1:** Sampling strata used in this study

Strata	Total size	Sampled	Fraction (%)
Microscopy+/PCR−	216	15	6.9
Microscopy−/PCR−	4169	301	7.2
Microscopy+/PCR+	1695	89	5.3
Microscopy−/PCR+	1057	61	5.8
Total	7137	466	6.5

### All-*Plasmodium* real-time PCR assay

Samples that initially tested negative by *pfldh* PCR underwent a real-time PCR assay that detects all species of *Plasmodium,* targeting the gene encoding the small sub-unit (18S) of the ribosomal RNA gene (heretofore referred to as “All *Plasmodium* qPCR”). Primer and probe sequences, reaction mixture and cycling conditions for this assay were previously published [12] with the exception that Probe Master qPCR Mix (Roche, Indianapolis, IN, USA) was used (Additional file 1: Table S1). Each sample was tested in duplicate. A sample was considered positive for *Plasmodium* if both of its replicates amplified or if one replicate amplified with a cycle threshold (C_T_) value lower than 38.

### Speciation by BLAST

Samples that tested positive in the All *Plasmodium* qPCR assay were speciated using Sanger sequencing and BLAST. DNA was amplified by nested PCR of *Plasmodium* 18S rDNA as previously described [13], with the outer primers rPLU1 and rPLU5 and the inner, genus-specific primers rPLU3 and rPLU4. Primer sequences and reaction conditions are listed in Additional file 1: Table S1.

Products from this nested PCR were Sanger sequenced (Eton Bioscience, Durham, NC, USA) and queried in the BLAST database (National Center for Biotechnology Information, Bethesda, MD, USA). If a sequence produced a result with at least 50 % query cover and 94 % identity, it was identified as the species with the best match. Otherwise, if a result produced 10 % query cover and 80 % identity, the cleanest part of the sequence, as determined by the authors, was resubmitted to BLAST and if this search fulfilled the initial criteria the species was defined. All sequences that did not produce such a match were labelled ‘indeterminate’ by this method.

### Speciation by species-specific nested PCR

Samples that were malaria-positive by either the *pfldh* qPCR or the All *Plasmodium* qPCR were tested using species-specific nested PCR as in [13]. The Round 1 reaction used the same primers as used in the genus-specific nested PCR but with Qiagen HotStarTaq Master Mix (Qiagen, Hilden, Germany) (Additional file 1: Table S1).

Separate Round 2 reactions were run for *P. falciparum* (primers rFAL1 and rFAL2), *P. malariae* (rMAL1 and rMAL2), and *P. ovale* (rOVA1 and rOVA2) (see Additional file 1: Table S1). The PCR products from each speciation reaction were analysed by gel electrophoresis on a 3 % agarose gel to determine positivity for each species in each sample. Samples that did not amplify for any of the three species were labelled ‘indeterminate’ by this method.

The species of each sample was determined based on results from nested PCR. If a sample was indeterminate by nested PCR, the BLAST result was used instead. If the BLAST result was also indeterminate, the sample was removed from the analysis.

### Analysis

Data were entered and analysed in Microsoft Excel 2007 (Microsoft, Redmond, WA, USA). Weighted prevalence were calculated as follows: for each stratum the proportion of positive samples in the sub-set was multiplied by the number of samples in the stratum. The sum of these was divided by the total number of samples.

## Results

### Study population

There were 7137 children under 5 years old with known *pfldh* PCR, microscopy and RDT results. Of these, a total of 466 samples (6.5 %) were chosen from the four strata listed in Table 1 for use in this study. A sample flow diagram is shown in Additional file 1: Figure S1.

### All *Plasmodium* qPCR

Among the 316 samples that were negative by *pfldh* PCR, 52 samples were malaria-positive by the All *Plasmodium* qPCR assay—nine microscopy-positive and 43 microscopy-negative. Including these and 150 samples that were positive by *pfldh* PCR, a total of 202 malaria-positive samples was analysed for speciation. Out of these, four (2.0 %) were indeterminate (Table 2), giving an analysable population of 198 malaria-positive samples and 462 total samples.

**Table 2:** Results of All *Plasmodium* qPCR and speciation of malaria-positive samples by stratum

	Microscopy+ *pfldh* PCR−	Microscopy− *pfldh* PCR−	Microscopy+ *pfldh* PCR+	Microscopy− *pfldh* PCR+	Total
*All Plasmodium qPCR*					
Negative	6	258	–	–	264
Positive	9	43	89	61	202
*Species-specific PCR*					
*P. falciparum* only	4	24	48	49	125
*P. malariae* only	0	5	0	0	5
*P. ovale* only	1	2	0	0	3
*P. falciparum* + *P. malariae*	4	2	23	5	34
*P. falciparum* + *P. ovale*	0	7	9	2	18
*P. falciparum* + *P. malariae* + *P. ovale*	0	0	9	4	13
*Mono-infections* *+* *Mixed*					
*P. falciparum* total	8	33	89	60	190
*P. malariae* total	4	7	32	9	52
*P. ovale* total	1	9	18	6	34
Indeterminate	0	3	0	1	4
Total	15	301	89	61	466

### Prevalence of *Plasmodium falciparum*

Of 198 malaria-positive samples, 190 (96.0 %) were positive for *P. falciparum*, giving a weighted prevalence in the survey sample set of 46.6 % (95 % CI 44.4–48.9 %) (Table 2). This is slightly higher than the prevalence of 38.6 % by the initial PCR test (*pfldh)* [10].

### Prevalence of *Plasmodium malariae* and *Plasmodium ovale*

**Fig. 1:**
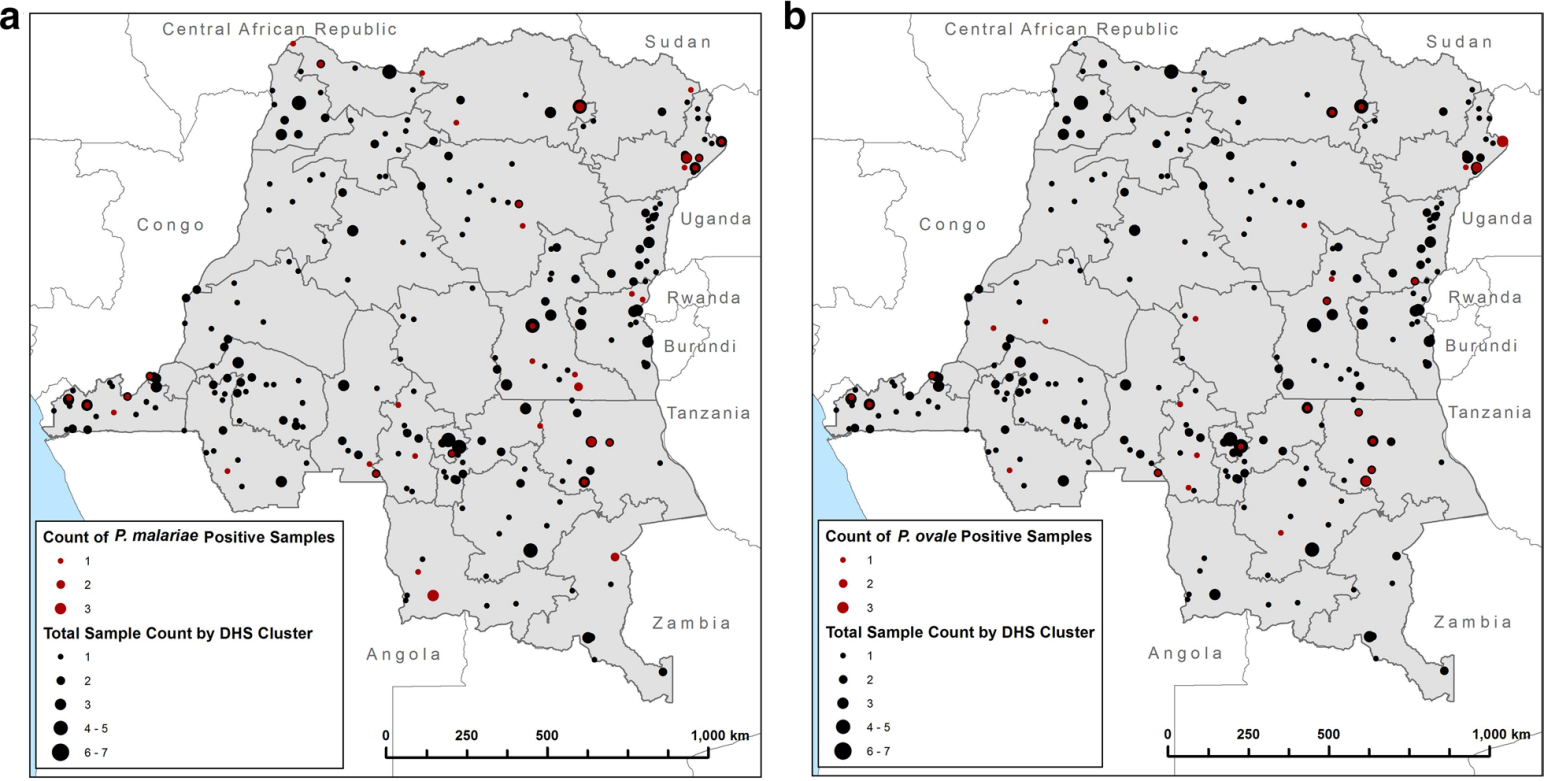
Geographical distribution of *Plasmodium malariae* (**a**) and *Plasmodium ovale* (**b**) cases by DHS cluster. For each cluster, the size of the *black dot* represents the number of cases tested (out of a total 462) and the size of the *red dot* represents the number of positive *P. malariae* (**a**) or *P. ovale* (**b**) cases

Of 198 samples, 52 (26.3 %) were positive for *P. malariae* and 34 (17.2 %) were positive for *P. ovale*, giving weighted prevalence of 12.9 % (95 % CI 10.0–15.9 %) and 8.3 % (95 % CI 5.7–10.8 %), respectively (Table 2). Geographical distributions of *P. malariae* and *P. ovale* infections are shown in Fig. 1. Both *P. malariae* and *P. ovale* infections are widely distributed. Eighty-nine percent of individuals with *P. malariae* or *P. ovale* infection were also infected with *P. falciparum*.

Five *P. malariae* mono-infections and three *P. ovale* mono-infections were found, giving weighted prevalence of 1.0 % (95 % CI 0.1–1.8 %) and 0.6 % (95 % CI 0–1.3 %), respectively. Of these eight non-*P. falciparum* mono-infections, six (75.0 %) were negative by RDT.

## Discussion

Using a sub-set of 462 samples from the large, cross-sectional DHS, the prevalence of *P. malariae* and *P. ovale* among children in the DRC was found to be 12.9 and 8.3 %, respectively, with widespread geographical distributions seen in both species. A recent study of children in Western Kasai, DRC found a similar prevalence of *P. malariae* (13.8 %) but a lower prevalence of *P. ovale* (2.4 %) [14], and another study of asymptomatic individuals in six provinces of the DRC found a much lower prevalence of *P. malariae* (1.0 %) [15]. In 2007 prevalence were 4.9 % for *P. malariae* and 0.6 % for *P. ovale* [16]. In general, prevalence found here are higher than those reported in other African countries for *P. malariae* [17–20] and *P.* ovale [17, 18, 21]. However, all of these differences could be due to normal geographic and temporal variations as well as differences in the PCR and sampling methods.

Mono-infections of *P. malariae* and *P. ovale* appear to be rare in the DRC. In this study, the mono-infection prevalence were only 1.0 and 0.6 %, respectively. In Western Kasai, there were no *P. malariae* or *P. ovale* mono-infections reported [14].

Because HRP2-based RDTs detect *P. falciparum* only, they can result in missed detection of *P. malariae* and *P. ovale* mono-infections. Of the eight mono-infections found here, six were negative by RDT. The remaining two were likely recently cleared *P. falciparum* infections in which the HRP2 antigen was still present, as it can remain in the blood stream for up to 1 month after parasite clearance [22]. Overall, the number of non-falciparum infections missed by the RDT was small as most such cases were co-infections with *P. falciparum*.

Of 316 samples that were initially negative by *pfldh* PCR, 52 amplified *P. falciparum* 18S rDNA by nested species-specific PCR. As a result, the PCR prevalence using both tests was higher (46.6 %) than found using a single test (38.6 %). This is likely because the 18S rDNA assay has a lower threshold of detection. Thus, caution must be used when comparing prevalence rates determined using different PCR and survey methodologies.

## Conclusions

*Plasmodium falciparum* remains the most prevalent species of malaria in the DRC, but *P. malariae* and *P. ovale* are endemic at a low rate. While RDTs have limitations, the results presented here suggest that the risk of missing malarial infections because they are mono-infections of *P. malariae* and *P. ovale* is low. However, the development of new RDTs to cover non-falciparum malaria will improve efforts at elimination.

## Additional file

10.1186/s12936-016-1409-0 PCR reaction and cycling conditions and sample flow diagram.

## Abbreviations

RDT: rapid diagnostic test
PCR: polymerase chain reaction
PfHRP2: *Plasmodium falciparum* histidine-rich protein II
pLDH: *Plasmodium* lactate dehydrogenase
DHS: demographic and health survey
DRC: Democratic Republic of the Congo
DBS: dried blood spots
