# Supplementary material for: Low prevalence of Plasmodium malariae and Plasmodium ovale mono-infections among children in the Democratic Republic of the Congo: a population-based, cross-sectional study
Source: Malar J. 2016 Jul 8;15:350. doi: 10.1186/s12936-016-1409-0 (PMC4938993; doi:10.1186/s12936-016-1409-0)
Supplement: Supplementary file 1 — 10.1186/s12936-016-1409-0 PCR reaction and cycling conditions and sample flow diagram. [file 12936_2016_1409_MOESM1_ESM.docx]

**Supplemental Table 1.** Primer and probe sequences, reaction mixtures and cycling conditions used in this study.

| Assay | Primer & Probe Sequences (5’ 🡪 3’) | Reaction Mixture | Cycling Conditions | Ref |
| --- | --- | --- | --- | --- |
| All *Plasmodia* qPCR | **Forward primer** GTT AAG GGA GTG AAG ACG ATC AGA TA  **Reverse primer** AAC CCA AAG ACT TTG ATT TCT CAT AAG  **Probe** VIC-TCG TAA TCT TAA CCA TAA AC-NFQ | FastStart Universal Probe Master Mix (Roche, Indianapolis, IN, USA)  1μM primers  200nM probe  4μL template | 95℃ 2 min  40 cycles:    95℃ 15 s    60℃ 1 min | [1] |
| Nested PCR Round 1 | **rPLU1** TCA AAG ATT AAG CCA TGC AAG TGA  **rPLU5** CCT GTT GTT GCC TTA AAC TCC | FastStart High Fidelity PCR System (Roche, Indianapolis, IN)  200nM primers  5μL template  HotStarTaq Master Mix (Qiagen, Hilden, Germany)  200nM primers  5μL template | 95℃ 2 min  35 cycles:    95℃ 30 s    53℃ 30 s    72℃ 1 min  72℃ 5 min  95℃ 15 min  35 cycles:    94℃ 1 min    50℃ 1 min    72℃ 1 min  72℃ 10 min | [2] |
| Nested PCR Round 2  (genus-specific) | **rPLU3** TTT TTA TAA GGA TAA CTA CGG AAA AGC TGT  **rPLU4** TAC CCG TCA TAG CCA TGT TAG GCC AAT ACC | FastStart High Fidelity PCR System (Roche, Indianapolis, IN)  200nM primers  5μL template | 95℃ 15 min  35 cycles:    94℃ 1 min    55℃ 1 min    72℃ 1 min  72℃ 10 min | [2] |
| Nested PCR Round 2  (falciparum) | **rFAL1** TTA AAC TGG TTT GGG AAA ACC AAA TAT ATT  **rFAL2** ACA CAA TGA ACT CAA TCA TGA CTA CCC GTC | HotStarTaq Master Mix (Qiagen, Hilden, Germany)  200nM primers  5μL template | 95℃ 15 min  35 cycles:    94℃ 1 min    62℃ 1 min    72℃ 1 min  72℃ 10 min | [2] |
| Nested PCR Round 2  (malariae) | **rMAL1** ATA ACA TAG TTG TAC GTT AAG AAT AAC CGC  **rMAL2** AAA ATT CCC ATG CAT AAA AAA TTA TAC AAA | HotStarTaq Master Mix (Qiagen, Hilden, Germany)  200nM primers  5μL template | 95℃ 15 min  35 cycles:    94℃ 1 min    58℃ 1 min    72℃ 1 min  72℃ 10 min | [2] |
| Nested PCR Round 2  (ovale) | **rOVA1** ATC TCT TTT GCT ATT TTT TAG TAT TGG AGA  **rOVA2** GGA AAA GGA CAC ATT AAT TGT ATC CTA GTG | HotStarTaq Master Mix (Qiagen, Hilden, Germany)  200nM primers  5μL template | 95℃ 15 min  35 cycles:    94℃ 1 min    62℃ 1 min    72℃ 1 min  72℃ 10 min | [2] |

**Supplemental Figure 1.** Sample flow diagram.

**
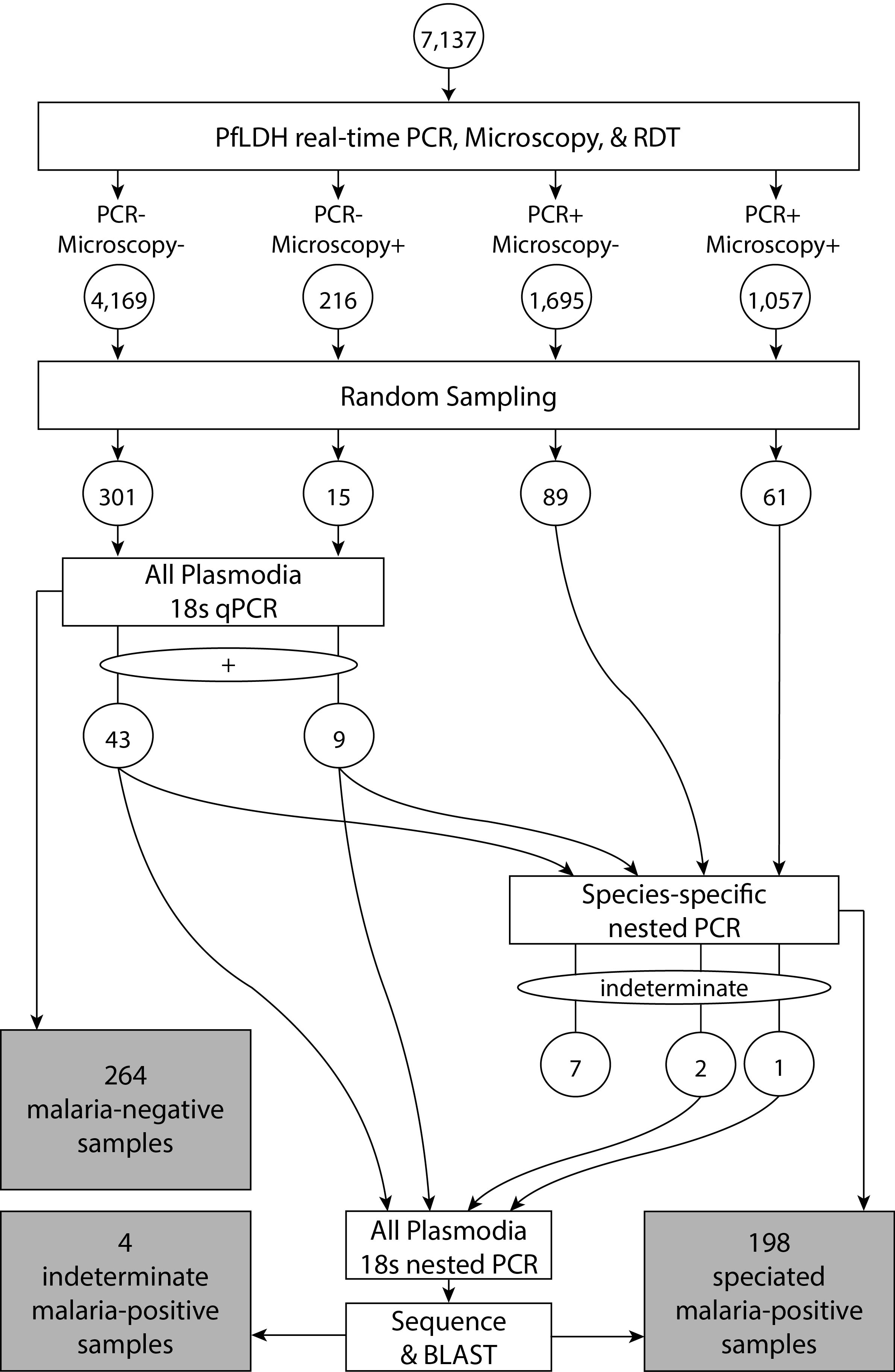
**

**References**

1. Taylor SM, Juliano JJ, Trottman PA, Griffin JB, Landis SH, Kitsa P, Tshefu AK, Meshnick SR: **High-throughput pooling and real-time PCR-based strategy for malaria detection.** *J Clin Microbiol* 2010, **48:**512-519.

2. Singh B, Bobogare A, Cox-Singh J, Snounou G, Abdullah MS, Rahman HA: **A genus- and species-specific nested polymerase chain reaction malaria detection assay for epidemiologic studies.** *Am J Trop Med Hyg* 1999, **60:**687-692.
